# Molecular epidemiological investigation of piroplasms carried by pet cats and dogs in an animal hospital in Guiyang, China

**DOI:** 10.3389/fmicb.2023.1266583

**Published:** 2023-10-12

**Authors:** Shengchun Wu, Jiao Meng, Fuxun Yu, Caomin Zhou, Bin Yang, Xingxing Chen, Guanghong Yang, Yi Sun, Wuchun Cao, Jiafu Jiang, Jiahong Wu, Lin Zhan

**Affiliations:** ^1^School of Public Health, The Key Laboratory of Environmental Pollution Monitoring and Disease Control, Ministry of Education, Guizhou Medical University, Guiyang, China; ^2^NHC Key Laboratory of Pulmonary Immune-related Diseases, Guizhou Provincial People’s Hospital, Guiyang, China; ^3^Renal Division, Department of Medicine, Guizhou Provincial People’s Hospital, Guizhou Provincial Institute of Nephritic and Urinary Disease, Guiyang, China; ^4^Guizhou Center for Disease Control and Prevention, Guiyang, China; ^5^Beijing Institute of Microbiology and Epidemiology, Academy of Military Medical Sciences, Beijing, China

**Keywords:** dog, cat, phylogenetic studies, piroplasms, *Theileria uilenbergi*, *Theileria luwenshuni*, *Colpodella*, Guizhou

## Abstract

Piroplasmosis is a zoonotic disease mainly caused by the *Babesia* and *Theileria* parasites. Piroplasmosis is often a subclinical infection in dogs and cats that is difficult to detect and is often suspected when clinical signs such as anemia are present. It has been reported to be prevalent in China. However, molecular evidence of the disease has not been reported in pet dogs and cats in Guiyang. In this study, we collected 307 anticoagulated blood samples from an animal hospital in the Wudang District of Guiyang during the period March 2021 to November 2021 and extracted DNA from the samples. The 18S rDNA gene was amplified using PCR, and the positive amplification product was sequenced. The sequences were then analyzed for homology and phylogeny. Of the 307 samples collected, 164 were feline and 143 were canine, with a total of 23 amplifying a target band of approximately 400 bp. The percentage of positives of piroplasms infection in pet cats was 4.27% (7/164), with the pathogens being *T. uilenbergi* (3) and *T. luwenshuni* (4). One *Colpodella* sp. and two undetermined species were also detected in the cat samples. The percentage of positives of piroplasms infection in pet dogs was 7.69% (11/143), with the pathogen being *T. uilenbergi* (11). One *Colpodella* sp. was also detected in the dog samples. The results confirmed that *T. uilenbergi* and *T. luwenshuni* are prevalent in pet cats and dogs in this area. In addition, the study found a rare zoonotic pathogen, *Colpodella* sp., in cats and dogs. Therefore, this study is expected to serve as a valuable reference for decision-making regarding animal health management and public health work.

## 1. Introduction

Piroplasms belong to the phylum Apicomplexa, class Piroplasmea, and order Piroplasmida and include the genera *Babesia* and *Theileria*. They are an order of protozoan parasites that live in macrophages, lymphocytes and red blood cells. Piroplasmosis is a human-animal infectious disease caused by piroplasms (Zhang and Li, 2010), with the main clinical symptoms being fever, anemia and swollen lymph nodes. *Babesia* is widely distributed around the world, and its onset often lacks typical symptoms, making it easy to misdiagnose or overlook. In recent years, the number of reported human cases has been increasing, with the United States, Canada and China having the most reported cases (Yang et al., 2021). The *Babesia* species that most often infect humans are *B. microti*, *B. venatorum*, *B. duncani*, and *B. divergens* (Ord and Lobo, 2015). *Theileria* species are mainly pathogenic to animals and cause serious losses in the livestock industry. Liu et al. (2010) detected *Theileria* sp. in the blood of a hospitalized patient in Suizhou City, Hubei Province (NCBI Accession: HQ844673.1). Although the paper has not yet been published, this finding suggests that *Theileria* sp. may be infectious to humans. Since there are relatively few studies on human *Theileria* infections in China, its study should not be neglected. At this time, none of the canine piroplasm species are zoonotic.

As living standards improve, pet ownership is becoming a normal part of life. Piroplasmosis in dogs and cats can be a chronic or subclinical infection, or it can be a severe acute disease in which the death of infected animals may occur (Irwin and Hutchinson, 1991). When piroplasmosis occurs a subclinical infection in dogs and cats, it is difficult to detect. It is often discovered due to clinical signs such as anemia, fever and lethargy. For example, 36 cases of *B. gibsoni* were seen in a veterinary hospital in Xi’an, and the signs and symptoms were mainly fever, yellow urine and decreased red blood cells and platelets (Feng et al., 2021). Blood transfusions can have an immediate effect, but they are expensive and there is often a shortage of blood available. Therefore, early identification of the pathogen facilitates the diagnosis and treatment of the disease, reducing the animal’s suffering as well as the medical burden.

However, there are no reports of piroplasmosis in dogs or cats in Guiyang, southwestern China. Therefore, this study aims to investigate the percentage of infections in pet cats and dogs in an animal hospital in Guiyang, and to provide a scientific basis for the prevention and control of piroplasms infections in pets in the region.

## 2. Materials and methods

### 2.1. Sample collection and ethics statement

From March 2021 to November 2021, the anticoagulated blood samples were collected from pet cats and dogs attending an animal hospital in Guiyang, China. The blood samples were stored in EDTA anticoagulation tubes, randomly numbered and then stored at 4°C for later use. For practical reasons, we were unable to obtain basic information about the animals (age, sex, etc.), geographic location of residence, and clinical data information.

This study was approved by the Animal Care Welfare Committee of Guizhou Medical University (Ethical approval number: 2305072). All animals were handled in accordance with the Animal Ethics Procedures and Guidelines of the People’s Republic of China. Informed consent was obtained from pet owners to acquire the anticoagulated blood samples from the animals.

### 2.2. Genomic DNA extraction

Nucleic acids were extracted from 200 μL of blood using the qEx-DNA/RNA Virus Kit (Xi’an Tianlong Science and Technology, Xi’an, China) according to the instructions, and then stored in a refrigerator at −20°C until PCR.

### 2.3. PCR amplification of the piroplasm 18S rDNA gene

PCR amplification was performed on all genomic DNA samples using the 18S rDNA gene nesting primers for piroplasm parasites (National Health Commission of the People’s Republic of China, 2017). A total volume of 25 μl was used for PCR amplification, which included 2.5 μl of 10 × PCR buffer, 2 μl of 2.5 mM dNTP Mixture, 0.5 μl of each primer (10 μM/L), 0.125 μl of Taq polymerase (5 U/μl) (Takara Biotechnology, China), 1 μl of extracted genomic DNA, and double-distilled water to fill the remainder. Genomic DNA from *B. bigemina* stored in our laboratory and sterile double-distilled water were used as positive and negative controls, respectively. The first round of nested PCR reaction procedures included pre-denaturation at 96°C for 2 min, denaturation at 94°C for 30 s, annealing at 54°C for 30 s, extension at 72°C for 40 s, a total of 35 cycles, and a final extension at 72°C for 5 min. The reaction conditions for the second round of Nestor PCR were the same as the reaction procedure for the first round, except that the annealing temperature was changed to 57°C. Finally, 5 μL of PCR products from all samples were taken and subjected to 1.5% agarose gels treated with 4S Green nucleotide stain (Sangon Biotech), and the results were observed using a gel imager (Bio-Rad).

### 2.4. Sequencing and phylogenetic analysis

The PCR products with amplified target bands were sent to BGI-Chongqing for bi-directional sequencing and sequence splicing. The obtained sequences were aligned with the sequences of registered genes in GenBank using the BLAST tool on the National Center for Biotechnology Information (NCBI) website. After identifying the species, multiple sequence alignments with related genes were conducted using DNAMAN (version 6.0, Lynnon Corporation, Canada) software and representative DNA sequences were taken. The reference sequences were also downloaded from the GenBank database. Representative sequences were selected for evolutionary tree construction, with *Plasmodium berghei* (NCBI Accession: AZ522148.1) and *P. falciparum* (NCBI Accession: DK896461.1) as outgroups. MEGA (version 7.0)^1^ software was used to construct a phylogenetic evolutionary tree using the neighbor-joining method and self-extension test 1,000 times for genetic evolutionary analysis.

## 3. Result

### 3.1. Prevalence of piroplasms in pets

A total of 307 anticoagulated blood samples were collected, including 164 pet cat samples and 143 pet dog samples. A total of 11 (6.7%) positive amplifications were obtained from the 164 pet cat blood samples, and 12 (8.4%) positives were obtained from 143 pet dog blood samples.

### 3.2. Genetic evolutionary analysis

A total of 22 target amplicon were successfully sequenced with BLAST homology matching, the piroplasms were sequenced as *T. uilenbergi* (14), *T. luwenshuni* (4) and *Colpodella* sp. (2), with the remaining two sequenced as other eukaryotes (Uncultured *Baldinia* and the Uncultured eukaryote). Specifically, pet cats were found to have *T. uilenbergi* (3), *T. luwenshuni* (4), *Colpodella* sp. (1), Uncultured *Baldinia* (1), and Uncultured eukaryote (1) (Table 1). All sequences were deposited in GenBank (Table 2). The sequences of *T. uilenbergi*, *T. luwenshuni*, *Colpodella* sp., Uncultured *Baldinia*, and Uncultured eukaryote genes detected in the cat samples were the closest to *T. uilenbergi* (NCBI Accession: MG940889.1), *T. luwenshuni* (NCBI Accession: MK685118.1), *Colpodella* sp. (NCBI Accession: JX624256.1), *Uncultured Baldinia clone* (NCBI Accession: MF685332.1), and *Uncultured eukaryote* (NCBI Accession: KU820642.1) in the GenBank database, respectively, while their sequence identities were 100.00, 100.00, 98.23, 99.26, and 98.69%, respectively. In addition, pet dog blood samples were found to have *T. uilenbergi* (11) and *Colpodella* sp. (1). Their gene sequences were closest in the GenBank database to *T. uilenbergi* (NCBI Accession: MG940889.1) and *Colpodella* sp. (NCBI Accession: OQ540589.1) with sequence identities of 100.00 and 94.99%, respectively. The sequence identity of the two *Colpodella* sp. (NCBI Accession: OR226256 and OR226258) was 81.71%, and compared with the reference strain *Colpodella* sp. ATCC50594 strain (NCBI Accession: AY142075) isolated from soil in the United States, the sequence identity was 81.77 and 83.00%, respectively. The sequence of *Colpodella* sp. from cat samples (NCBI Accession: OR226256) was only 79.69%∼83.14% when compared with *Colpodella* sp. strains from suspected clinical cases in China (NCBI Accession: KT364261 and GQ411073), but the sequence identity with another suspected clinical case from China (NCBI Accession: MF594625) was as high as 99.60%. When the sequence of *Colpodella* sp. from dog samples (NCBI Accession: OR226258) was compared with the sequences of *Colpodella* sp. strains from suspected clinical cases (NCBI Accession: KT364261, GQ411073, and MF594625), the sequence identity was only 79.01∼84.39%.

**TABLE 1:** Different pets carry different numbers of protozoa.

Animals	sample size	*Theileria uilenbergi*	*Theileria luwenshuni*	*Colpodella sp.*	Uncultured *Baldinia*	Uncultured eukaryote
Pet cats	164	3	4	1	1	1
Pet dogs	143	11	—	1	—	—
Total	307	14	4	2	1	1

**TABLE 2:** The NCBI Accession of this study gene sequences in GenBank.

NCBI accession	Species	Animals
OR016205	*T. uilenbergi*	Pet cats
OR016425	*T. luwenshuni*	Pet cats
OR226256	*Colpodella* sp.	Pet cats
OR226257	Uncultured *Baldinia*	Pet cats
OR226255	Uncultured eukaryote	Pet cats
OR016208	*T. uilenbergi*	Pet dogs
OR226258	*Colpodella* sp.	Pet dogs

**FIGURE 1:**
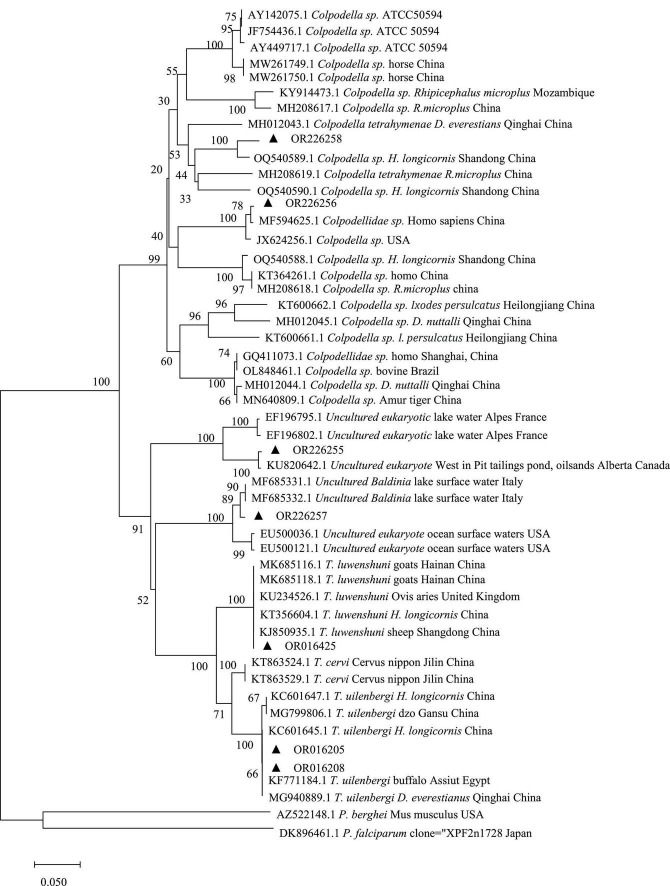
Genetic evolutionary analysis of the 18S rDNA piroplasm gene in pet cats and dogs. The 18S rRNA sequences obtained in this study were indicated with black triangles.

The gene sequences of pet cats (NCBI Accession: OR016205) and pet dogs (NCBI Accession: OR016208) formed a clade with high homology to the sequences of *T. uilenbergi* infecting yaks in the Gansu region, *Dermacentor everestianus* in the Qinghai region, and *Haemaphysalis longicornis* in the Beijing region (NCBI Accession: MG799806.1, MG940889.1, and KC601647.1), with 99.73%∼100.00% homology. The gene sequences of pet cats (NCBI Accession: OR016425) were homopolymerized into a clade with 100.00% homology to the sequences of *T. luwenshuni* infecting sheep in Shandong (NCBI Accession: KJ850935.1) and the United Kingdom (NCBI Accession: KU234526.1). The above *Theileria* gene sequences were clustered with *T. cervi* sequences (NCBI Accession: KT863524.1 and KT863529.1) infecting Sika deer in the Jilin region of China, all of which were *T.* sp. The pet cat gene sequences (NCBI Accession: OR226256) clustered into a single clade with *Colpodellidae* sp. (NCBI Accession: MF594625.1) detected in the blood of a patient with relapsing fever in China, with 99.60% homology. However, pet cat gene sequences (NCBI Accession: OR226256) were distant from *Colpodella* sp. gene sequences detected in ticks (NCBI Accession: OQ540589), horses (NCBI Accession: MW261749), cattle (NCBI Accession: OL848461), and tigers (NCBI Accession: MN640809). The pet dog gene sequences (NCBI Accession: OR226258) clustered into a clade with *Colpodella* sp. (NCBI Accession: OQ540589.1) detected in *H. longicornis* from Yiyuan County, Shandong, China, with a homology of 94.99%. The pet dog gene sequences (NCBI Accession: OR226258) were distant from *Colpodella* sp. gene sequences detected in humans (NCBI Accession: MF594625), horses (NCBI Accession: MW261749), cattle (NCBI Accession: OL848461) and tigers (NCBI Accession: MN640809). In addition, the studied sequences (NCBI Accession: OR226257 and OR226255) were clustered separately and not assigned to a eukaryotic group, occupying an evolutionary position between *Colpodella* sp. and *Theileria* (Figure 1).

## 4. Discussion

The percentage of positives of piroplasms infection in pet dogs in this study was 7.69% (11/143), which was higher than the prevalence of canine-origin piroplasm infections reported in Türkiye (0.13%, 2/757) (Aktas et al., 2015) and China’s Hunan Province (4.30%, 5/115) (Wang J. et al., 2020). This may be related to the different geographic locations where the samples were collected, the sensitivity of the diagnostic technique used, and the different clinical statuses of the dogs studied. To the best of our knowledge, this study is the first to report the detection of *T. uilenbergi* in the blood of pet dogs in China. The common hosts of *T. uilenbergi* are sheep and deer (Mans et al., 2015), and host infections may present with fever, diarrhea, respiratory distress and enlarged lymph nodes. However, there are no reports on the pathology and symptoms of *T. uilenbergi* in dogs, and the clinical significance of this is not known. The vectors of *T. uilenbergi* have been confirmed to be *H. longicornis* and *H. qinghaiensis* (Li et al., 2009), and Xiang et al. (2022) reported that *H. longicornis* is the dominant tick species in Guizhou, which increases the likelihood of a *T. uilenbergi* epidemic in the area. The main piroplasms reported to infect dogs in China and abroad are *B. canis*, *B. vogeli*, *B. rossi*, *B. conradae*, *B. vulpes*, and *B. gibsoni* of the genus *Babesia* (Irwin, 2010; He et al., 2017; Niu et al., 2017). Although *Babesia* was not detected in the pet dog samples in this study, *Colpodella* sp. (NCBI Accession: OR226258) was detected in one dog sample, and its pathogenicity requires further investigation. The sequence from the pet dog (NCBI Accession: OR226258) was in close proximity to *Colpodella* sp. (NCBI Accession: MH012043, OQ540589, MH208619, and OQ540590) detected in ticks (Figure 1). This result suggests that ticks that may carry *Colpodella* sp. and could be screened for possible *Colpodella* sp. at this site in the future. The Wudang District of Guiyang has many natural scenic spots, and there are a number of parks with dense vegetation and rich ecology distributed around this hospital. When pet owners walk their dogs in the park, their dogs may be bitten by ticks when brushing against vegetation and may be infected with pathogens carried by ticks during the biting process, posing a threat to the animals’ health.

The percentage of positives of blood piroplasms in pet cats in this study was 4.27% (7/164), which was lower than the 8.00% (2/25) infection rate reported in Hunan Province (Wang J. et al., 2020). In this study, the pet cat pathogens were more diverse, containing not only *T. uilenbergi*, but also *T. luwenshuni*, *Colpodella* sp. and two unknown species of gene sequences. Reports of piroplasm infections in domestic cats and wild felines include the species such as *B. felis*, *B. cati*, *B. leo*, *B. hongkongensis*, *B. gibsoni*, and *Cytauxzoon* sp. (Hartmann et al., 2013; Palmer et al., 2022; Yin et al., 2022), but there have been no reports on the detection of *T. uilenbergi* and *T. luwenshuni* in blood samples from pet cats. This may be related to the fact that piroplasm in cats is often an asymptomatic infection (Wong et al., 2012), resulting in a lack of research and underreporting. *T. uilenbergi* and *T. luwenshuni* are common *Theileria* in China and are the more pathogenic species (Hao, 2020). They are often found in mixed infections. Studies have reported that *T. luwenshuni* can infect a wide range of host animals, such as goats, sheep, deer and sheepdogs (Ge et al., 2012; Li et al., 2014; Gholami et al., 2016), and it is evident that it may have a wider distribution range. For example, Zhong et al. (2019) and Wang K. L. et al. (2020) investigated the infection of cattle and sheep with *T. luwenshuni* in Aba Prefecture, Sichuan Province and Linyou County, Shanxi Province and found that the infection rates were as high as 75.00 and 61.20%, respectively. The rare *Colpodella* sp., a tick-borne pathogen that has been detected in Qinghai Province (Hu et al., 2022), was also detected in the blood samples of pet cats. *Colpodella* sp. is also a zoonotic pathogen, with the first case of a *Colpodella* sp.-like pathogen infecting a human found in Kunming, Yuan et al. (2012). *Colpodella* sp. was also detected in horses from Jingxi and Napo, Guangxi (Xu et al., 2022; Zhou et al., 2022), as well as being recently detected in the blood of Amur tigers (Chiu et al., 2022). Cui (2013) also amplified *Colpodella* sp. sequences in the blood of febrile patients and in the cerebrospinal fluid of in-patients with neurological symptoms (Jiang et al., 2018). The cat sample *Colpodella* sp. sequence (NCBI Accession: OR226256) clustered with the reference strain ATCC50594 and was closer to the gene sequences of the currently published patients (NCBI Accession: MF594625), with a higher sequence identity (99.60%). The possibility of zoonotic disease transmission to humans in this region cannot be excluded. In this study, it should be noted that *Colpodella* is not a piroplasm. *Colpodella* is a sister group to an apicomplexan clade (Kuvardina et al., 2002). The initial aim of the study was to investigate blood carriage of piroplasms in cats and dogs with 18S rRNA amplification sequencing, but we inadvertently discovered *Colpodella*, which is a significant finding. Therefore, it is not unusual that *Colpodella* was amplified unintentionally with these common primers, which is similar to what has been observed in previous studies (Jiang et al., 2018; Xu et al., 2022). The *Colpodella* sp. pathogen and whether it can infect humans remains controversial and requires further confirmation. The taxonomic statuses of the gene sequences of two unknown species also need to be confirmed.

The PCR molecular biology technique used in this study addresses the morphological difficulty of distinguishing parasites in the blood when the quantity is low. It is able to accurately detect the presence of pathogens. However, there are some limitations to the study, which failed to collect basic information regarding the pet dogs and cats during the sample collection process and failed to analyze the epidemiological characteristics of the hosts in a comprehensive manner. According to the current detection results, the tick-borne pathogens *Theileria* and *Colpodella* sp. were found in the blood samples of dogs and cats. The data suggest that *Theileria* and *Colpodella* sp. were existent in dogs and cats in Guizhou. The *Theileria* and *Colpodella* sp. found in the study are different in their treatments. At the present stage, there is no highly efficient drug for the treatment of piroplasmosis caused by *T. uilenbergi* and *T. luwenshuni*, and the more commonly used drugs include Imidocarb, Diminazene Aceturate, Acriflavine, Atovaquone, Azithromycin, Clindamycin, Quinine, etc. (Hao, 2020). *Colpodella* has been reported in fewer cases in host animals and humans. The paucity of case reports makes it difficult to draw conclusions regarding treatment. For example, the patient reported by Yuan et al. showed some common features with *Babesia* cases and responded well to treatment with Atovaquone and Azithromycin (Yuan et al., 2012). The patient reported by Jiang et al. (2018) exhibited neurological symptoms and was treated with Doxycycline. Neculicioiu et al. (2021) found that a combination regimen of Ceftriaxone and Metronidazole was effective against urinary contamination due to *Colpodella* sp.

In conclusion, this study is the first report of pet dogs and cats infected with *T. uilenbergi*, *T. luwenshuni*, and *Colpodella* sp. in Guiyang, southwestern China, which provides scientific data for the diagnosis of the common piroplasmosis in pet dogs as we as decision-making in the management of animal health and public health.

## Data availability statement

The data presented in the study are deposited in the NCBI repository, accession numbers OR016205, OR016425, OR226256, OR226257, OR226255, OR016208, and OR226258.

## Ethics statement

The animal studies were approved by the Animal Care Welfare Committee of Guizhou Medical University. The studies were conducted in accordance with the local legislation and institutional requirements. Written informed consent was obtained from the owners for the participation of their animals in this study.

## Author contributions

SW: Conceptualization, Data curation, Formal analysis, Investigation, Validation, Visualization, Writing – original draft, Writing – review and editing, Project administration. JM: Investigation, Supervision, Writing – review and editing, Project administration. FY: Funding acquisition, Supervision, Writing – review and editing, Project administration. CZ: Supervision, Writing – review and editing, Project administration. BY: Supervision, Writing – review and editing, Project administration. XC: Supervision, Writing – review and editing, Project administration. GY: Supervision, Writing – review and editing. YS: Supervision, Writing – review and editing. WC: Supervision, Writing – review and editing. JJ: Project administration, Supervision, Writing – review and editing, Validation. JW: Funding acquisition, Project administration, Supervision, Writing – review and editing. LZ: Funding acquisition, Investigation, Project administration, Supervision, Writing – review and editing, Methodology.

## Footnotes

1
http://www.megasoftware.net/
